# Far-Red Turn-On Fluorogenic Probe for Versatile Detection of Inorganic Polyphosphates

**DOI:** 10.1021/jacs.6c14037

**Published:** 2026-10-02

**Authors:** Kenji Torii, Ru̅ta Gerasimaitė, Gražvydas Lukinavičius

**Affiliations:** Chromatin Labeling and Imaging Group, Department of NanoBiophotonics, 632136 Max Planck Institute for Multidisciplinary Sciences, Am Fassberg 11, Göttingen 37077, Germany

## Abstract

Inorganic polyphosphate (polyP) is a ubiquitous phosphate biopolymer involved in diverse cellular processes. Despite its significance, selective detection of polyP remains challenging because of its simple and highly charged structure. Here, we report a far-red fluorogenic turn-on chemosensor for selective polyP detection and imaging, SiX-DPA-Zn. It integrates a silicon-xanthene fluorophore with a Zn­(II)-coordinated 2,2’-dipicolylamine recognition unit. Replacing the bridging oxygen in the xanthene scaffold with silicon shifts fluorescence into the far-red region and confers up to 100-fold selectivity for inorganic polyP over other phosphate-containing biomolecules, including nucleic acids and nucleoside di­(tri)­phosphates. SiX-DPA-Zn enables quantitative detection of polyP at micromolar concentrations in microplate assays and stains a broad range of polyP species, starting from tripolyphosphate, in polyacrylamide gels. In HEK293 cells expressing *Escherichia coli* polyphosphate kinase 1, the probe visualizes intracellular polyP and enables quantitative analysis of its distribution relative to nuclear proteins—fibrillarin and nucleolin. Stimulated emission depletion (STED) microscopy further resolves subdiffraction-sized polyP granules within nuclear polyP aggregates. Together, these results establish SiX-DPA-Zn as a versatile chemical tool for quantitative polyP detection across biochemical assays and advanced fluorescence imaging.

## Graphical abstract

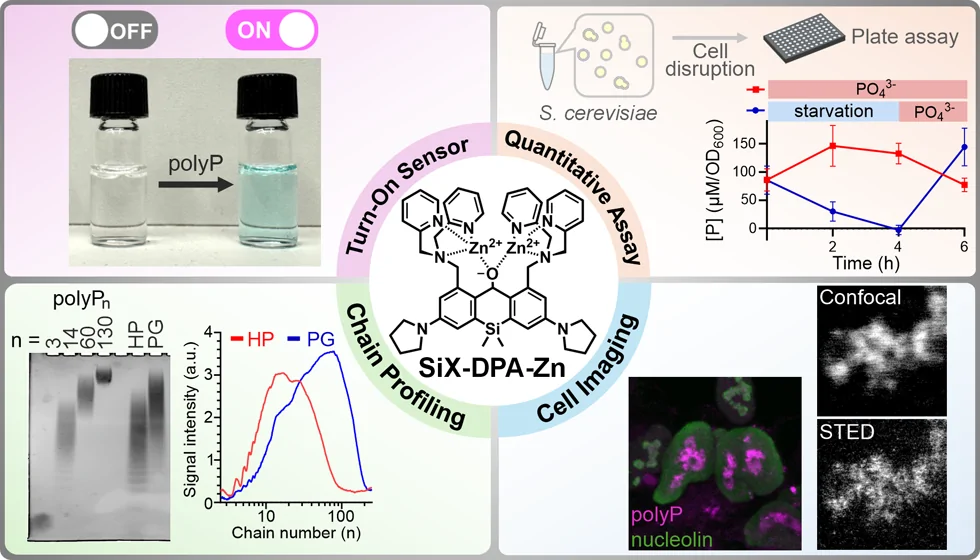

## Introduction

Inorganic polyphosphate (polyP), composed of chains of tens to thousands of orthophosphates, plays numerous roles in all forms of life, ranging from bacteria to mammals.^1–4^ For example, it functions as an energy and phosphate storage,^5–7^ metal chelator,^8,9^ buffer to alkaline,^10^ protein-protecting chaperone,^11–13^ regulator of gene expression,^14^ and protector against DNA damage.^15^ PolyP is also associated with blood clotting^16^ and inflammation.^17,18^ More recently, some studies have implicated polyP in neurodegeneration, including Parkinson’s and Alzheimer’s diseases, and amyotrophic lateral sclerosis.^19,20^ Given the broad and rapidly growing interest in polyP research, the development of chemical sensors capable of selectively detecting and visualizing polyP in biological systems has emerged as an increasingly important direction in the field.^4,20,21^

Despite the high demand for polyP sensors, only a few probes that can sense polyP have been reported so far. The first example is 4’,6-diamidino-2-phenylindole (DAPI), which is commonly used as a DNA stain (emission at around 450 nm). It is also used as a polyP sensor, detecting the red-shifted yellow emission (525–550 nm) that occurs upon binding to polyP.^22,23^ However, DAPI exhibits similar yellow emission after binding to RNA (around 500 nm)^24,25^ and inositol pyrophosphates (around 550 nm),^26^ which reduces its specificity for polyP. JC-D dyes are recognized as second-generation polyP sensors, aiming at higher specificity than DAPI.^27^ Nevertheless, JC-D dyes exhibit strongly reduced fluorescence in physiological salt conditions due to weakened ionic dye–polyphosphate interactions, which severely limits reliable intracellular polyP staining.^27^ Most recently, an excited-state intramolecular proton transfer (ESIPT)-based fluorescent probe for polyphosphate detection has been reported, enabling sensitive measurements in solution.^28^ However, its applicability in biological systems remains unverified. Currently, the most widely used approach for visualizing polyP in mammalian cells employs the polyphosphate-binding domain (PPBD), which binds polyP with high affinity and specificity.^29–32^ It is typically fused with an epitope tag (e.g., Xpress tag) for immunostaining using primary and fluorophore-conjugated secondary antibodies. In addition to this procedure being labor-intensive and time-consuming, the multiple fixation and washing steps may cause loss or redistribution of short-chain polyP. Hence, there is a strong need for the development of a chemosensor that enables facile detection of polyP in biological systems.

Several studies have shown that binuclear 2,2′-dipicolylamine (DPA)–metal (particularly Zn^2+^) complexes function as phosphate-binding motifs and have been widely used in chemosensors for various phosphate-containing substances,^33–37^ nucleoside polyphosphates,^38–45^ and pyrophosphate (PPi).^41,46–49^ We hypothesized that coupling a silicon-substituted xanthene fluorophore with a DPA–Zn^2+^ recognition motif would provide an effective platform for polyP sensing by tuning the electronic properties of the fluorophore and facilitating a turn-on response upon interaction with highly charged inorganic polyphosphates.

Thus, we synthesized a novel inorganic polyP-selective, far-red-emissive probe designated as SiX-DPA-Zn. Notably, SiX-DPA-Zn exhibits micromolar affinity and up to 100-fold selectivity for polyP over nucleic acids and nucleoside di­(tri)­phosphates (ATP, ADP, GTP, CTP, and UTP). This enables monitoring enzymatic polyP degradation by calf intestinal alkaline phosphatase (ALP) and *E. coli* exopolyphosphatase (PPX), and quantitative determination of polyP in *Saccharomyces cerevisiae* lysates in microplate-based assays. In addition, SiX-DPA-Zn allows visualization of polyP in polyacrylamide gels, including short-chain species such as tripolyphosphate, which are poorly detected by conventional dyes such as DAPI, JC-D7, and toluidine blue-O (TB-O). Importantly, the probe is applicable to cellular imaging, enabling visualization of polyP in mammalian cells expressing *E. coli* polyphosphate kinase 1 (*Ec*PPK1) by confocal and STED microscopy.

## Results and Discussion

### Design and Synthesis of SiX-DPA (−Zn)

In designing the sensor, we focused on a nucleoside polyphosphate turn-on probe incorporating two 2,2’-dipicolylamine (DPA)–Zn^2+^ complexes onto xanthene (hereafter referred to as X-DPA-Zn), as reported by Hamachi et al.^39,40^ In X-DPA-Zn, the oxygen atom bridged by the two DPA-Zn units forms a covalent bond with the C9 position of the xanthene scaffold, rendering the molecule non-fluorescent. Interaction with nucleoside di­(tri)­phosphates, such as ADP and ATP, disrupts this C–O bond, leading to strong fluorescence.^39^

Encouraged by this intriguing property of the DPA-Zn complex, we hypothesized that inorganic polyP could also be recognized using a similar approach. To this end, we designed SiX-DPA-Zn, operating in the far-red fluorescence channel, which is less subject to interference by autofluorescence of cell components than blue and green channels employed by DAPI and other polyP probes. SiX-DPA-Zn consists of two DPA-Zn units and a silicon-substituted xanthene scaffold, with each DPA-Zn unit directly incorporated at the 1- and 8-positions of the fluorophore (Figure 1A). SiX-DPA-Zn was synthesized in 10 steps (9 pots) to afford the product in high purity (>99%) with an overall yield of 1.1% (Figure 1B). The most critical step is the conjugation of DPA to the silicon-substituted xanthene. We found that a one-pot reaction, combining an Appel reaction followed by S_N_2 amination, afforded a good yield. This is likely attributable to the high instability of the dibrominated intermediate, which undergoes substantial decomposition during reaction workup and purification. Careful handling is also required during the purification of SiX-DPA, as it is easily oxidized in methanol. When purified using methanol, we observed non-negligible byproducts in LC–MS with a mass increases of 16 and 34 Da, indicating the addition of one oxygen atom and an additional water molecule at the meso position (C9) of the xanthene core (Figure S1). Other studies have also reported that the Si-pyronin structure is unstable during purification^50^ or when stored in methanol.^51^ The oxidation of SiX-DPA was successfully prevented by using acetonitrile during purification. After purification and lyophilization, the product purity was confirmed to be greater than 99% based on absorbance measurements at both 254 and 650 nm (Figure S1). The purified compound was stored in dimethyl sulfoxide (DMSO) at −80 °C for subsequent experiments. Detailed synthetic procedures and complete characterization data for all intermediates and the final compound are provided in the Supporting Information.

**Figure 1:**
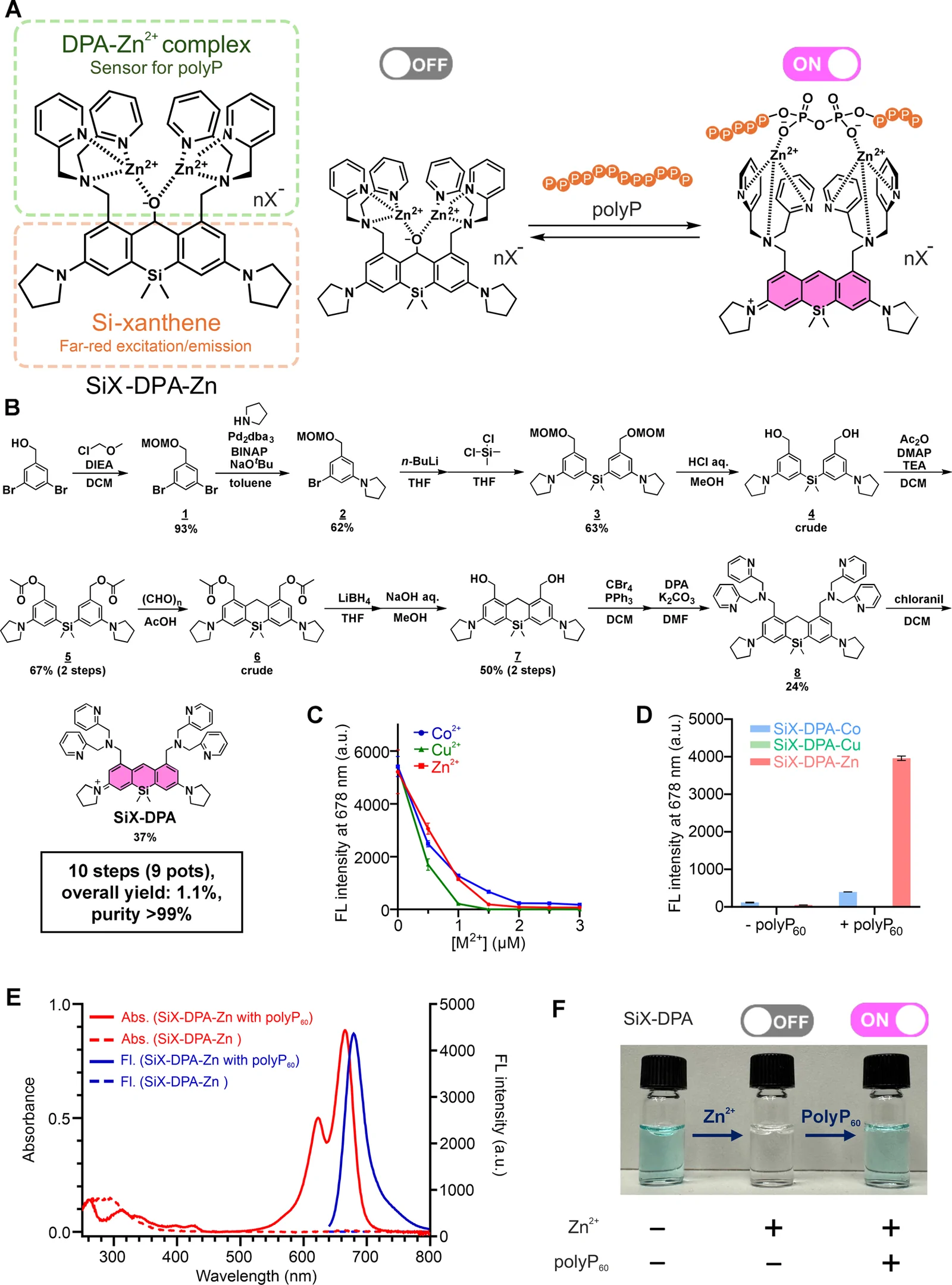
Molecular design, synthesis, and photophysical characterization of SiX-DPA. (A) Molecular design and proposed polyphosphate (polyP) sensing mechanism of SiX-DPA-Zn. (B) Synthetic route to SiX-DPA. (C) Fluorescence titration of SiX-DPA (1.0 μM) with ZnCl_2_, CuCl_2_, or CoCl_2_ in 20 mM HEPES buffer (pH 7.5). (D) Fluorescence response of SiX-DPA-Co, SiX-DPA-Cu, and SiX-DPA-Zn (1.0 μM) to the addition of 100 μM polyP_60_. Data represented as mean ± s.d. of three independent replicates. (E) Absorption and fluorescence spectra of SiX-DPA-Zn (10 μM for absorbance measurement; 1.0 μM for fluorescence measurement) in the absence and presence of polyP_60_ (100 equiv) in 20 mM HEPES buffer (pH 7.5). (F) Color transition of 10 μM SiX-DPA solutions. From left to right: SiX-DPA alone, SiX-DPA with Zn^2+^, and SiX-DPA with Zn^2+^ plus polyP_60_ (1 mM).

### Photophysical Properties of SiX-DPA and SiX-DPA-M

Purified SiX-DPA exhibits strong far-red absorption (*λ*_ex,max_ = 666 nm, *ε* = 1.1x10^5^ M^–1^cm^–1^) and fluorescence emission (*λ*_em,max_ = 678 nm, QY = 0.24, τ = 0.23 and 2.52 ns) in aqueous solution (Figure S2 and Table S1). We evaluated the effect of various divalent and trivalent metal ions and found that the addition of two equivalents of Co^2+^, Cu^2+^, or Zn^2+^ resulted in substantial fluorescence quenching (Figures 1C and S3). We propose that it is initiated by covalent bond formation between the C9 position of the xanthene scaffold and an oxygen atom bridging two DPA–metal ion units, resulting in disruption of the π-conjugated xanthene system.

We next examined the pH dependence of the fluorescence response and observed that the quenched state (SiX-DPA-M) remains stable at pH > 7.0 (Figure S4). Both SiX-DPA-Zn and SiX-DPA-Co exhibit fluorescence recovery under acidic conditions starting from pH < 6.0, with complete recovery observed at pH 4.0. This is likely due to protonation of the DPA moiety, which decreases its affinity for zinc and cobalt ions.^52,53^ We also observed a gradual decrease in fluorescence above pH 8.5 (Figure S4). This can be attributed to nucleophilic attack of the hydroxyl group (OH^–^) at the meso position, followed by subsequent oxidation. Therefore, we concluded that the fluorescence turn-off system operates effectively within the pH range of 7.0–8.0, which encompasses physiological conditions. Unless otherwise noted, all *in vitro* polyP detection experiments were performed in HEPES buffer at pH 7.5.

The complexation of SiX-DPA with Zn^2+^ was further analyzed by LC–MS (Figure S5). The analysis revealed distinct predominant species depending on the solvent, indicating that the complex exists in an equilibrium between two different states (Figure S5A). In HEPES buffer, the major ion was assigned to a SiX-DPA complex containing two Zn­(II) ions and a single oxygen anion (Figure S5B). High-resolution mass spectrometry revealed a peak at m/z 486.142, assigned to [SiX-DPA-Zn­(O^–^)­(HCOO^–^)]^2+^ (calculated m/z 486.141; Figure S6), with an isotopic distribution characteristic of a species containing two Zn atoms. However, in DMSO, the major ion corresponded to SiX-DPA, suggesting that Zn­(II) and SiX-DPA form a relatively labile and dynamically exchanging coordination complex (Figure S5C). NMR spectra recorded in DMSO-*d*_6_ showed pronounced broadening of proton resonances and the disappearance of several ^1^H and ^13^C signals near the Zn­(II)-binding sites, which can be interpreted as reflecting a dynamically exchanging Zn­(II) complex. Thus, the SiX-DPA–Zn^2+^complex exhibits solvent-dependent coordination behavior. The non-aqua species (“non-aqua SiX-DPA-Zn”) is favored in DMSO, whereas the oxygen-ligated species (SiX-DPA-Zn) is favored in aqueous buffer (Figure S5A). Unless otherwise stated, SiX-DPA-Zn refers to the binuclear Zn complex containing an oxygen ligand at the meso position, as depicted in Figure 1A.

Next, we examined whether SiX-DPA-Zn fluorescence intensity changes can be used for polyP detection. In the absence of polyP, the absorption and fluorescence signals of SiX-DPA-Zn were too weak to allow clear identification of the corresponding spectral maxima, whereas SiX-DPA-Zn exhibited markedly higher absorbance and fluorescence response to polyP_60_ than the other metal complexes (*λ*_ex,max_ = 666 nm and *λ*_em,max_ = 679 nm) after adding polyP_60_ (Figure 1D, E). Its photophysical properties were similar to the metal-free SiX-DPA precursor, although it showed a threefold lower fluorescence quantum yield and a shorter fluorescence lifetime (Table S1). The color change of the SiX-DPA solution upon addition of Zn^2+^ and its subsequent recovery following supplementation with polyP_60_ could be readily observed, as illustrated in Figure 1F.

We were not able to detect the SiX-DPA-Zn/polyP complex by LC–MS. The acidic pH 3.5 of the LC-MS buffer, which weakens Zn^2+^ coordination, and the possibly dynamic nature of the complex are the likely reasons preventing its direct detection. However, the existence of such a complex can be inferred from the differences in quantum yield and fluorescence lifetime observed between free SiX-DPA and SiX-DPA-Zn in the presence of polyP (Table S1).

### Determination of SiX-DPA-Zn Affinity to Various Phosphate-Containing Compounds

After identifying the potential of SiX-DPA-Zn to detect polyP, we investigated its specificity by comparing affinity for polyP and other phosphate-containing compounds (Figure 2A). As analytes, we examined polyP of different chain lengths (average lengths of 14 (polyP_14_), 60 (polyP_60_), and 130 (polyP_130_)), orthophosphate (Pi); pyrophosphate (PPi); triphosphate (PPPi, same as polyP_3_) and organic polyphosphates—adenosine diphosphate (ADP), adenosine triphosphate (ATP), guanosine triphosphate (GTP), cytidine triphosphate (CTP), and uridine triphosphate (UTP)—and nucleic acids (calf thymus DNA and total yeast RNA). To examine the possibility of using X-DPA-Zn as a polyP sensor in the green channel, we also investigated it in parallel.

**Figure 2:**
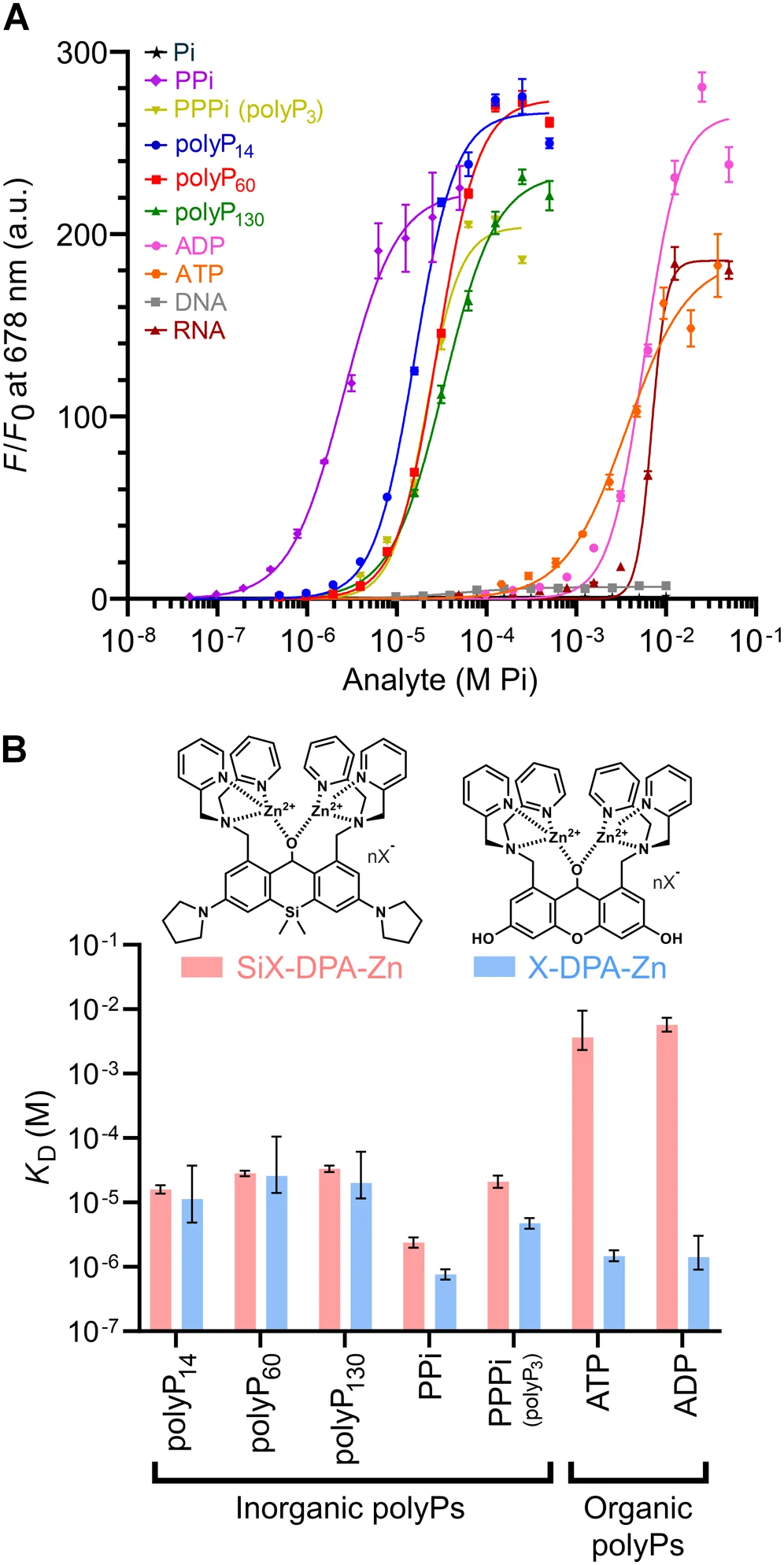
Binding affinity and selectivity of SiX-DPA-Zn toward phosphate-containing compounds. (A) Fluorescence titration curves of SiX-DPA-Zn (1.0 μM) with polyPs of different chain lengths (14, 60, and 130), Pi, PPi, PPPi (polyP_3_), ATP, ADP, DNA, and RNA. *λ*_ex/em_ = 620/678 nm. Fluorescence responses are shown as normalized intensities (*F*/*F*_0_), where F_0_ represents the fluorescence intensity in the absence of analyte. Concentrations of all phosphate-containing compounds are expressed as phosphate equivalents (M Pi) to allow straightforward comparison between different species. (B) Comparison of apparent dissociation constants (*K*_D_) of SiX-DPA-Zn and X-DPA-Zn toward polyPs, PPi, PPPi (polyP_3_), ATP, and ADP. *K*_D_ is calculated from the Hill equation fitting. All data are represented as mean ± s.d. of three independent replicates.

Titration experiments with SiX-DPA-Zn demonstrated significant differences in affinity between inorganic polyPs and other phosphate-containing substances. PPi showed the highest affinity of 2.4 μM, while longer polyPs of all chain lengths (polyP_3_, polyP_14_, polyP_60_, and polyP_130_) exhibited slightly lower affinity, with the chain length having little effect (*K*_D_ = 21, 16, 28, and 33 μM, respectively). ADP, ATP, other nucleoside triphosphates, and RNA showed considerably lower affinity (*K*_D_ > 1 mM) (Table S2). No fluorescence increase was observed upon addition of orthophosphate (Pi) and DNA.

X-DPA-Zn showed an affinity for all polyPs, similar to that of SiX-DPA-Zn, and negligible binding to nucleic acids. As previously reported,^39^ it showed high affinity (*K*_D_ < 1 μM) for ATP and ADP (Figures 2B and S7). Moreover, SiX-DPA-Zn exhibited a larger fluorescence enhancement (*F*_max_/*F*_0_ = ∼200-fold) upon binding to polyPs, compared to X-DPA-Zn (*F*_max_/*F*_0_ = ∼10-fold) (Tables S2 and S3). These results suggest that SiX-DPA-Zn can produce better contrast, is more specific for polyP, and therefore is a better polyP sensor than X-DPA-Zn.

We hypothesize that the increased selectivity of SiX-DPA-Zn compared to X-DPA-Zn for polyP arises from altered electronic properties and improved scaffold preorganization, which together reduce binding to ATP and ADP. Replacement of the bridging oxygen at the 10-position with silicon changes the electronic structure of the fluorophore due to the lower electronegativity and higher polarizability of silicon. This modification alters charge distribution and may influence Zn­(II)–DPA coordination and phosphate recognition. In addition, substitution with silicon leads to a small (∼0.3 Å) decrease in the distance between the 1- and 8-substituent positions, consistent with the larger atomic radius of silicon and longer Si–C bonds (Figure S8). This subtle geometric change may affect the preorganization of the binding sites.

### Monitoring polyP Degradation *In Vitro* by Polyphosphatases

To examine the utility of the fluorogenic SiX-DPA-Zn response for polyP quantification *in vitro*, we built calibration curves and found a linear relationship between polyP concentration and fluorescence in the 0–10 μM range (Figure S9). For SiX-DPA-Zn, the detection limit (0.5 μM polyP) was higher than that of DAPI^23^ and JC-D7 ^54^ (0.125 μM) (Figures S9 and S10).

Several points should be considered when performing quantitative analysis of polyP using SiX-DPA-Zn. First, the apparent affinity of SiX-DPA-Zn for polyP decreases in the presence of high concentrations of phosphate, likely due to competition for probe binding (Figure S11). Second, ethylenediaminetetraacetic acid (EDTA), which is often used to quench metal-dependent enzymatic reactions, can deprive SiX-DPA-Zn of Zn^2+^with apparent affinity of 9 μM (Figure S12). This leads to a fluorescence increase that might cause the overestimation of the polyP concentration. Third, fluorescence intensity is influenced by magnesium ions, which are often present in biological buffers. This effect is presumably due to magnesium ions forming complexes with polyP,^55,56^ thereby hindering its interaction with SiX-DPA-Zn. We also examined the effect of proteins, using BSA as a standard protein and found that BSA did not impair the interaction between SiX-DPA-Zn and polyP (Figure S11). Therefore, we strongly recommend constructing calibration curves under identical buffer conditions, particularly with the same magnesium concentration, and avoiding the use of metal chelating agents (Figure S13). We also note that 0.1% Triton X-100 was added to HEPES buffer to maintain probe dispersion and ensure reliable fluorescence measurements in the following experiments (Figure 3).

**Figure 3:**
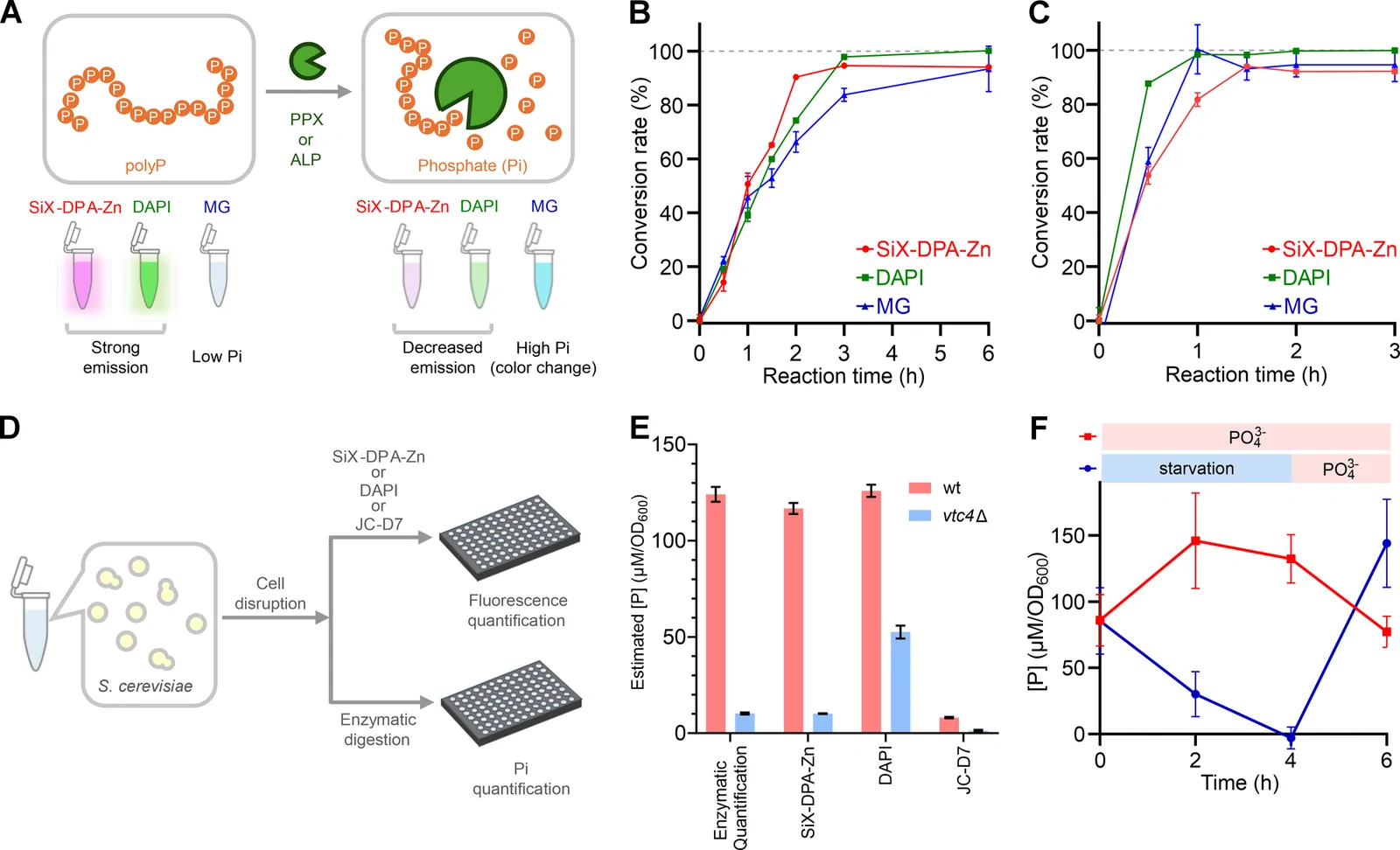
PolyP quantification using SiX-DPA-Zn. **(A**) Schematic illustration of enzymatic polyP degradation and its analysis using SiX-DPA-Zn or DAPI fluorescence, and the malachite green (MG) colorimetric assay for free phosphate. 1 mM polyP_130_ was incubated with (**B**) *E. coli* exopolyphosphatase (PPX, 500 ng mL^–1^) or (**C**) calf intestine alkaline phosphatase (ALP, 50 μg mL^–1^), aliquots were taken at the indicated time points and polyP-dye fluorescence or released Pi was measured. Before fluorescence measurements, reaction mixtures were diluted 50-fold with 20 mM HEPES buffer. In the case of SiX-DPA-Zn, the solution was supplemented with 0.1% Triton X-100 in order to prevent probe aggregation. **(D)** Workflow of polyP quantification in yeast *S. cerevisiae* extracts. **(E)** polyP concentration in wt and polyP-deficient *vtc4*Δ yeast determined by SiX-DPA-Zn, DAPI, and JC-D7 fluorescence, or by enzymatic quantification. Note that the apparently high polyP levels found in the *vtc4*Δ strain by the DAPI assay are an artefact caused by co-isolated RNA (see Figure S15). **(F)** Monitoring polyP depletion and overplus in yeast during the phosphate starvation–resupply cycle. Red and blue plot lines indicate Pi-starved and control cultures, respectively. After 4 h of starvation, 10 mM KH_2_PO_4_ was added to the phosphate-depleted culture. All data represented as mean ± s.d. of three independent biological replicates.

After establishing conditions for polyP quantification with SiX-DPA-Zn, we monitored polyP digestion by *E. coli* exopolyphosphatase (PPX) and alkaline phosphatase (ALP)^57^ in the end-point assay (Figure 3A). We prepared a polyP_130_ solution, incubated it with each enzyme in the appropriate reaction buffer, and quantified the polyP levels at defined time points. The performance of SiX-DPA-Zn was compared with that of DAPI, which was used as a reference method for polyP quantification (Figure S14A, B, D and E). Possible interference from the phosphate generated in the reaction and the magnesium required for enzyme activity was counteracted by diluting the reaction mixture before probe addition and producing calibration curves with the identical buffer composition as the samples (Figure S13). At the same time, we determined the concentration of released inorganic phosphate with the Malachite Green (MG) assay^58^ (Figure S14C and F). All three methods yielded similar reaction time courses (Figure 3B and C), indicating that SiX-DPA-Zn can be used to track polyP concentrations like the DAPI and MG assays.

### Quantitative Analysis of polyP in *Saccharomyces cerevisiae* Cell Extracts

To evaluate the applicability of SiX-DPA-Zn to analyze polyP from natural sources, we measured the concentration of polyP in yeast extracts (Figure 3D). As a control, we used the *vtc4*Δ strain that lacks polyphosphate synthase Vtc4 and contains negligible levels of polyP.^59^ SiX-DPA-Zn was compared with other polyP-sensitive dyes (DAPI and JC-D7) and with the enzymatic gold-standard method for polyP quantification, in which polyP is hydrolyzed by polyphosphatase and pyrophosphatase and the concentration of the released Pi is measured by a colorimetric reaction.^60,61^ After cell disruption with glass beads, the supernatant (cell lysate) was diluted to the appropriate concentration range for each assay (final concentration of 2–20 μM for the dye-based methods and 20–200 μM for the enzymatic method). The polyP concentration was then determined using calibration curves generated for each method (Figure S9). The enzymatic assay and SiX-DPA-Zn found similar polyP levels in wild-type (wt) yeast and low residual levels in the *vtc4*Δ mutant (Figure 3E). Consistent with its high sensitivity to salt, JC-D7 heavily underestimated the polyP concentration.^27^ PolyP levels measured with DAPI in the wt strain were similar; however, this method reported unexpectedly high polyP in *vtc4*Δ cells. RNase treatment of the lysates reduced the fluorescent signal of DAPI in both samples and led to underestimation of polyP levels in wt yeast (Figure S15A). This underestimation may be attributable to the lower sensitivity of DAPI toward short-chain polyP and/or variations in DAPI–polyP fluorescence caused by the composition and ionic strength of the cellular lysate.^62,63^ Importantly, RNase treatment had no effect on the SiX-DPA-Zn signal, suggesting negligible interference from RNA (Figure S15B).

Next, we used SiX-DPA-Zn to monitor polyP degradation and resynthesis during the phosphate-starvation-replenishment cycle (Figures 3F and S16). PolyP levels dropped to undetectable levels during 4 h of starvation and recovered to even higher levels within 2 h upon phosphate addition to the medium.

Altogether, these results demonstrate that SiX-DPA-Zn enables the rapid and precise quantification of polyP concentration in *S. cerevisiae* without the need for enzymes and with no interference from RNA.

### Staining polyP in Polyacrylamide Gels

Assessment of polyP size distribution by polyacrylamide gel electrophoresis (PAGE) is an essential technique in investigating polyP metabolism, biological functions, and the activity of polyP-synthesizing and -degrading enzymes. We compared the performance of SiX-DPA-Zn in staining polyP in gels to that of other commonly used dyes—DAPI (positive and negative detection), JC-D7, and toluidine blue (TB-O).^64^ To this end, we ran four identical 30% gels with 10 nmol/well of different polyP species and stained them with the four dyes. To stain the gel with SiX-DPA-Zn, we incubated the gel with a 10 μM probe in HEPES buffer supplemented with 20% ethanol and 2% glycerol for 30 min. We used the published protocols for other dyes (see the Methods section for details). Upon excitation with 630 nm light, the SiX-DPA-Zn-stained gel revealed a fluorescent polyP smear (Figure 4A) with a chain length distribution similar to, but not identical to, that produced by other methods (Figure 4B). The most striking difference was the ability of SiX-DPA-Zn to stain polyPs as short as polyP_3_, which are not detectable with DAPI, JC-D7, or TB-O. This result is broadly consistent with the previously reported reduced sensitivity of DAPI and TB-O toward short-chain polyP.^21^ To the best of our knowledge, JC-D7 has not been used for staining polyPs in gels before. In our hands, JC-D7 revealed a similar polyP size distribution to DAPI and TB-O and also failed to detect short-chain polyPs.

**Figure 4:**
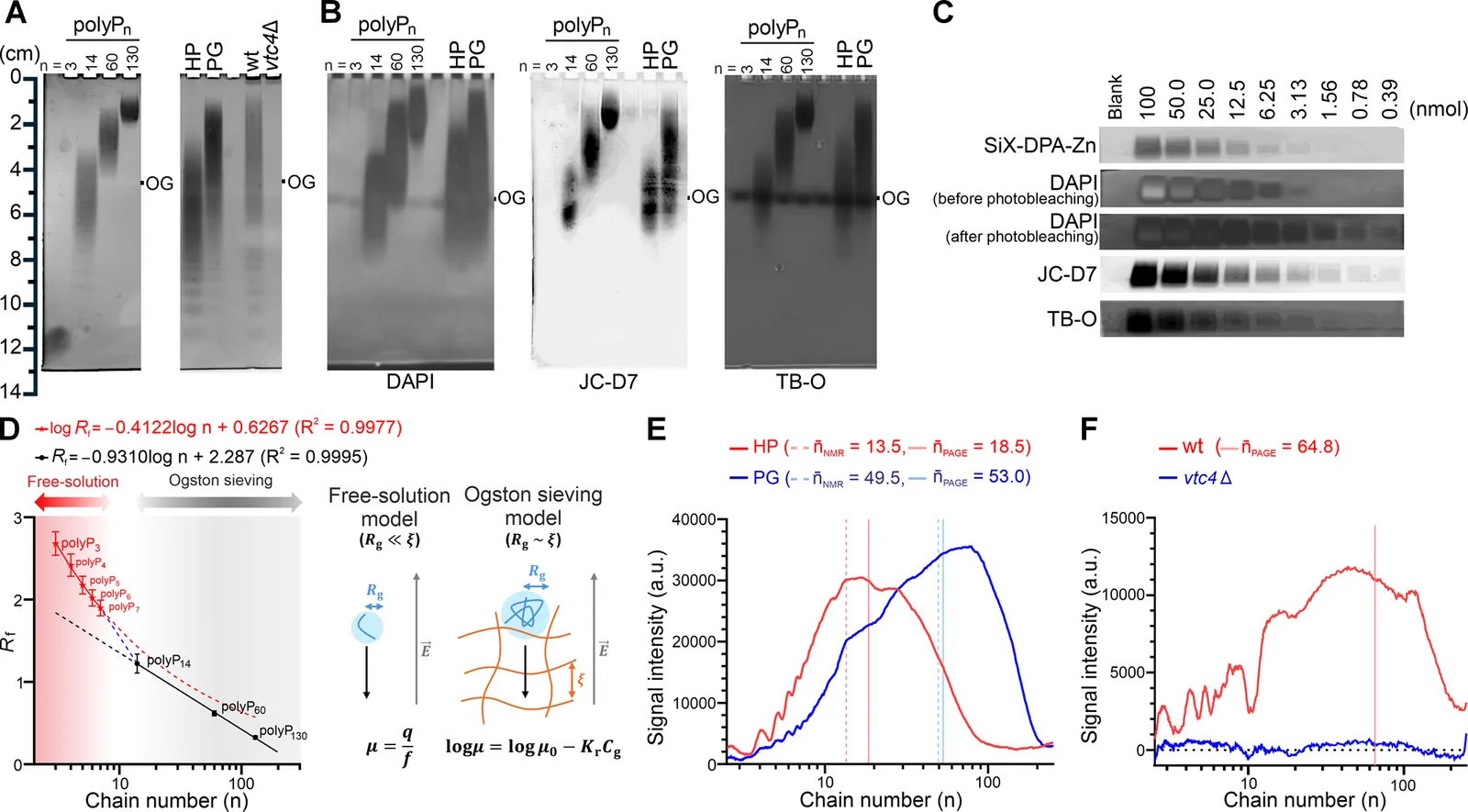
PolyP analysis by polyacrylamide gel electrophoresis (PAGE). **(A)** Detection of separated polyP species by SiX-DPA-Zn staining in 30% PAGE gel. Synthetic polyPs, hexametaphosphate (HP), phosphate glass (PG), and RNA/polyP extracts from *S. cerevisiae* wt and *vtc4*Δ strains were separated by gel electrophoresis. OG is Orange G dye. (B) Comparison of staining performance for resolved polyP species in 30% PAGE gels using DAPI (negative staining), JC-D7, and toluidine blue (TB-O). (C) Sensitivity of polyP detection in gels. Various amounts of polyP_60_ (0.39–100 nmol) were separated in 15% PAGE, and gels were stained with SiX-DPA-Zn, DAPI (before/after photobleaching), JC-D7, and TB-O. The images were compressed vertically for visualization. Full gels are in Figure S18. (D) The relationship between relative mobility (*R*_f_) and chain number of polyP (n) determined using polyP_3_ and the resolved polyphosphate bands (polyP_4–7_) of HP (red line). Linear regression of the logarithm of chain number (n) versus relative mobility (*R*_f_) was performed using polyP_14_, polyP_60_, and polyP_130_ (black line). The blue dashed line connects the *R_f_* values of polyP_7_ and polyP_14_ plotted against log n. The *R*_f_ values are expressed as the mean ± standard deviation from three independent experiments. Right panel shows a schematic comparison of free-solution transport to long-chain Ogston sieving regimes. Free-solution mobility is governed by the effective charge (q) and friction coefficient (*f)*, related to the polymer radius of gyration (*R*_g_). Sieving mobility is governed by the retardation coefficient (*K*_r_) and gel concentration (*C*_g_), related to the gel mesh size (ξ). **(E, F)** Estimated chain-length distributions of (E) HP and PG, and (F) polyP extracts from *S. cerevisiae* wt and *vtc4*Δ cells calibrated using polyP_3_, polyP_14_, polyP_60_, and polyP_130_ standards. Dashed pale red and blue lines indicate average chain lengths determined by ^31^P NMR, while corresponding solid lines indicate values estimated from PAGE analysis.

SiX-DPA-Zn successfully stained polyP extracted from *S. cerevisiae*. A strong polyP signal was observed in samples from wt cells, whereas no signal was detected in the *vtc4*Δ strain (Figure 4A). Weak, slowly migrating discrete bands present in both samples completely disappeared upon RNase treatment, indicating that, similarly to DAPI, SiX-DPA-Zn can detect co-isolated RNA (Figure S17). This appears to be at odds with measurements performed in solution, where interference from RNA was minimal, and with the high apparent *K*_*D*_ of the SiX-DPA-Zn interaction with RNA. However, conditions in gels are substantially different. More material is loaded on the gel than is used in solution measurements. During electrophoresis, RNA migrates as a compact band, where a sufficiently high concentration may be reached for it to be stained by SiX-DPA-Zn despite low affinity.

The sensitivity of polyP detection by SiX-DPA-Zn staining was comparable to DAPI before photobleaching and to TB-O (∼1.6 nmol), but lower than DAPI after photobleaching and JC-D7 (<0.4 nmol) (Figures 4C and S18). Despite this, SiX-DPA-Zn offers a clear advantage of fast and simple staining procedure without prolonged washing and photobleaching. Notably, it enables direct visualization of polyPs with a degree of polymerization below 10, which has not been previously achieved except for methods requiring acid hydrolysis prior to methyl green staining.^65^

The ability to detect polyP_3_ provides an unambiguous reference and enables direct determination of the exact chain lengths of very short polyPs. Thereby we resolved individual polyP_3–7_ bands in the HP sample (Figure 4A). The relative mobility (*R*_f_) of each band was calculated as the migration distance relative to the Orange G (OG) dye front. *R*_f_ values were then plotted against polyP chain length based on the Ogston sieving model (Figure 4D).^66–68^ Long-chain polyPs followed the expected sieving behavior. In contrast, short-chain polyPs (polyP_3–7_) required a different fitting model because their migration is better described by free-solution behavior, where the gel mesh has little effect. Both calibration curves showed excellent linearity, with R^2^ > 0.995, based on at least three independent gels (Figure 4D and Tables S4 and S5).

Based on the regressions obtained in Figure 4D, the distributions of the degree of polymerization for the polyphosphate samples HP and PG were determined and compared to those obtained by ^31^P NMR (Figure 4E). We found average chain lengths (*n̅*_PAGE_) of 18.5 for HP and 53.0 for PG. The average chain lengths of HP (*n̅*_NMR_ =13.5) and PG (*n̅*_NMR_ = 49.5) were determined by ^31^P NMR (Figure S19).^21,69^ The values from PAGE were slightly higher than those from NMR. This difference arises because fluorescence intensity reflects the total amount of bound probe and thus the mass of polyP rather than the number of chains. As a result, PAGE analysis yields a weight-average distribution, whereas NMR provides a number-average value, leading to an apparent shift toward longer chain lengths. The chain-length distribution of the polyP extract from *S. cerevisiae* was also analyzed in the same manner, yielding an average

Chain length of 64.8 (Figure 4F) The signal in the ^31^P NMR spectrum was too weak to determine the average chain length of this sample. This demonstrates that our method is well suited for analyzing a broad range of polyP samples, including those not easily accessible by ^31^P NMR.

### polyP Visualization in Mammalian Cells

To assess the feasibility of using SiX-DPA-Zn for visualizing polyP by fluorescence microscopy, we stained a recently developed model cell line expressing *E. coli* polyphosphate kinase 1 (*Ec*PPK1) under the tetracycline repressor (TetR).^32^ Endogenous polyP levels in mammalian cells are low and, thus, challenging to detect. In this system, doxycycline induces expression of the*E. coli* transgene, which leads to dramatically increased polyP levels. After 24 h of induction, cells were fixed, stained with SiX-DPA-Zn for 20 min, and imaged with a confocal fluorescence microscope. Doxycycline-induced cells showed strong punctate fluorescence signals in the nucleus, while little staining could be detected in noninduced controls (Figure 5A). A similar staining pattern was observed with red-shifted, polyP-specific DAPI fluorescence (Figure S20A). We also tested JC-D7, a commercially available polyP-sensitive fluorophore.^27^ However, no detectable fluorescence signal was observed in either induced or noninduced cells (Figure S21), consistent with previous reports showing limited applicability of JC-D-series dyes in mammalian cells.^32^

**Figure 5:**
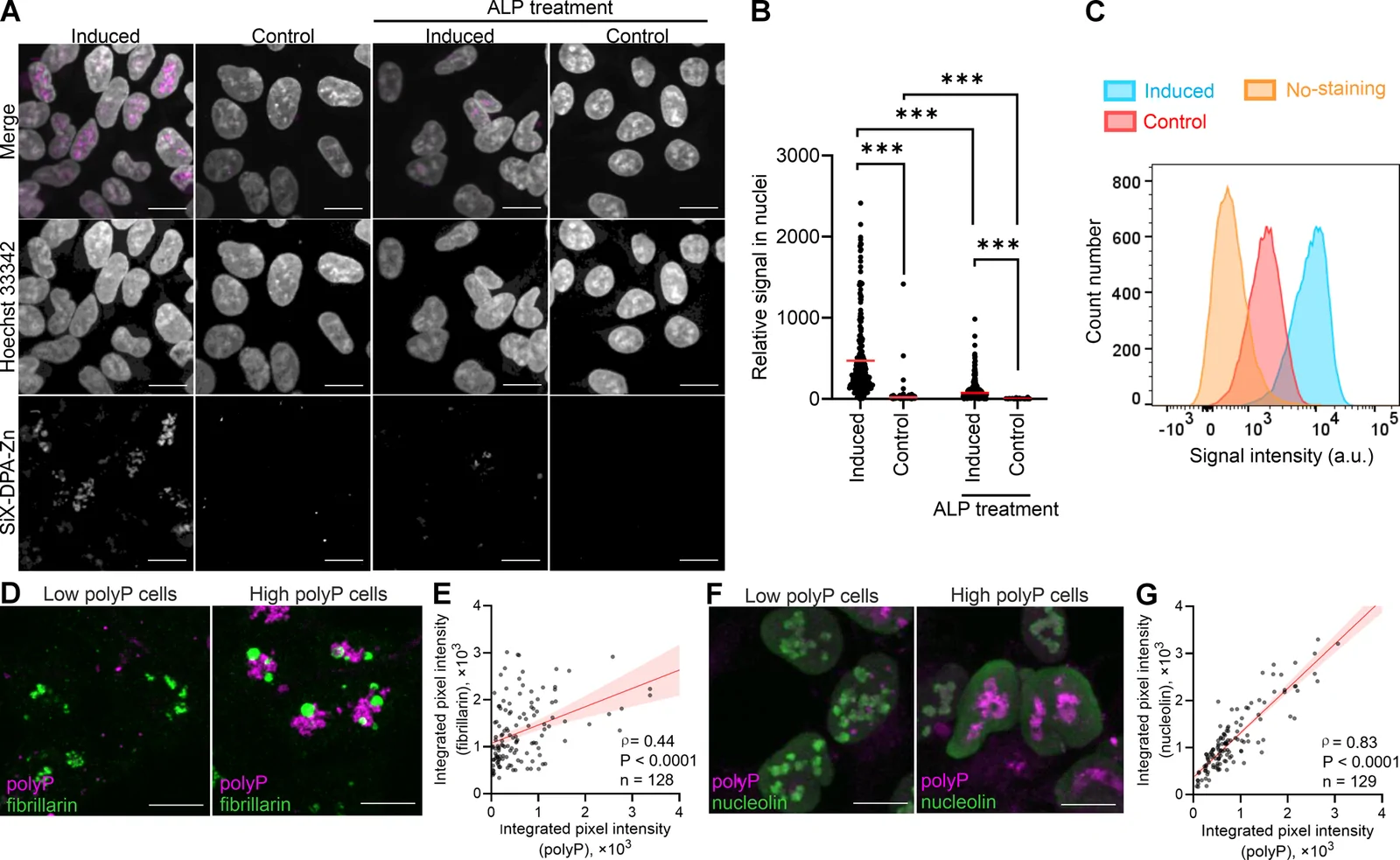
PolyP analysis in TREx-PPK1 cells using SiX-DPA-Zn. **(A)** Confocal images of TREx-PPK1 cells with and without doxycycline induction for 24 h. PolyP was stained with SiX-DPA-Zn (5.0 μM, magenta) and nuclei with Hoechst 33342 (1.0 μM, gray). PolyP depletion was achieved by alkaline phosphatase (ALP, 1.0 mg mL^–1^) treatment for 1 h prior to analysis. Scale bars: 20 μm. (B) Quantification of nuclear polyP intensity from imaging data. The number of analyzed nuclei was 235 (induced, no ALP), 236 (control, no ALP), 205 (induced, with ALP), and 186 (control, with ALP). The red line represents the mean. The nucleus area was detected using the Hoechst 33342 signal. *** - *p* < 0.0001. (C) Flow cytometric analysis of single-cell polyP levels (excitation 633 nm, emission 670/14 nm). Cell numbers analyzed: 27,601 (induced), 26,841 (control), and 31,763 (unstained). (D) Co-staining of polyP (magenta) with fibrillarin (green) in induced cells showing low and high polyP levels. Scale bars: 10 μm. (E) Spearman correlation analysis between polyP and fibrillarin signal intensities (n = 128 cells). The red line represents the linear regression, and the shaded area indicates the 95% confidence interval. (F) Co-staining of polyP (magenta) with nucleolin (green) in induced cells showing low and high polyP levels. Scale bars: 10 μm. (G) Spearman’s correlation analysis between polyP and nucleolin signal intensities (n = 129 cells). The red line represents the linear regression, and the shaded area indicates the 95% confidence interval. (**A**, **D**, **F**)­Maximum intensity projections of confocal z-stacks.

Pretreatment of samples with alkaline phosphatase (ALP) before staining significantly reduced the fluorescence signal, indicating that the nuclear signals detected by both DAPI and SiX-DPA-Zn reflect intracellular polyP (Figures 5A and S20A). Quantitative analysis of individual nuclei staining confirmed this conclusion for both dyes (Figures 5B and S20B).

The SiX-DPA-Zn signal was photostable and allowed three-dimensional (3D) imaging of polyP distribution throughout the nuclear volume.

We often observed brightly stained 3D polyP aggregates of granular structure, reminiscent of polyP-rich domains previously detected by SEM^32^ (Video S1). These results demonstrate that SiX-DPA-Zn is a highly effective polyP-staining probe with low background fluorescence and a high signal-to-noise ratio, enabling accurate detection of polyP in cells.

Next, we applied the probe to fluorescence-activated cell sorting (FACS) analysis to examine large cell populations. Consistent with the microscopy results, doxycycline-induced cells showed a strong increase in SiX-DPA-Zn fluorescence compared with control cells (Figure 5C), demonstrating selective detection of polyP at the single-cell level. In contrast, FACS analysis using DAPI showed only minimal differences between induced and control cells, likely because of background fluorescence from DAPI bound to DNA and RNA (Figure S22). These results highlight the advantage of the fluorogenic turn-on response of SiX-DPA-Zn for the flow cytometric analysis of cellular polyP.

From a spectral perspective, DAPI emits across both blue and green channels, whereas SiX-DPA-Zn is restricted to a single far-red channel. This spectral separation is particularly advantageous for studying intracellular polyP in complex cellular contexts by using multicolor imaging.

First, we investigated whether heterogeneity of polyP levels is caused by variable levels of *Ec*PPK1 expression. Co-staining cells with SiX-DPA-Zn and anti-Myc tag antibody and quantifying both signals revealed two cell populations. Among cells with low to moderate *Ec*PPK1 expression, the SiX-DPA-Zn and anti-Myc signals showed a clear positive correlation (Spearman’s ρ = 0.42, Figure S23). In contrast, cells with high *Ec*PPK1 expression showed no significant correlation between the two signals (Spearman’s ρ = −0.01, Figure S23) and did not accumulate polyP. These results suggest that *Ec*PPK1 levels contribute to cellular polyP accumulation but do not fully determine polyP load. Several other factors may contribute to this behavior. For example, high-level expression of *Ec*PPK1 may promote protein aggregation or reduce the fraction of functionally active enzyme. In addition, the cellular metabolic capacity to support polyP synthesis may become limiting at high *Ec*PPK1 expression.

A previous study characterizing the TREx-PPK1 cell line described disorganization of the nucleolus and upregulation of nucleolar components, including fibrillarin and nucleolin, upon induction of *Ec*PPK1 expression.^32^ The latter conclusion was based mainly on protein levels measured by Western blotting, which reflect population-level analysis. Taking advantage of multicolor imaging and heterogeneous polyP load in the cell population, we were able to confirm this observation at the single-cell level and directly correlate polyP with nucleolin and fibrillarin (Figures 5D–G, S24 and S25). To this end, we co-stained cells with antibodies against the respective proteins and SiX-DPA-Zn and quantified both components in individual cells. Cells with higher polyP levels showed increased fibrillarin staining (Figures 5D and S24). Spearman’s rank correlation analysis revealed a moderate positive correlation (ρ = 0.44, P < 0.0001, n = 128) (Figure 5E). An even stronger positive correlation was observed between polyP and nucleolin levels (ρ = 0.83, P < 0.0001, n = 129) (Figure 5G).

### Subcellular Distribution of polyP Revealed by Super-Resolution Microscopy

SiX-DPA-Zn is compatible with high-resolution STED imaging because its far-red-to-near-infrared fluorescence can be efficiently depleted using a 775-nm STED laser (Figures 6A, B, and S26). STED images revealed finer details of polyP aggregates with features smaller than the light-diffraction limit and beyond the resolution of conventional confocal microscopy (Figures 6C and S26). The average size (FWHM) of polyP granules was 138 ± 82 nm, as measured by STED microscopy, compared with 743 ± 333 nm determined by confocal microscopy (Figure 6C).

**Figure 6:**
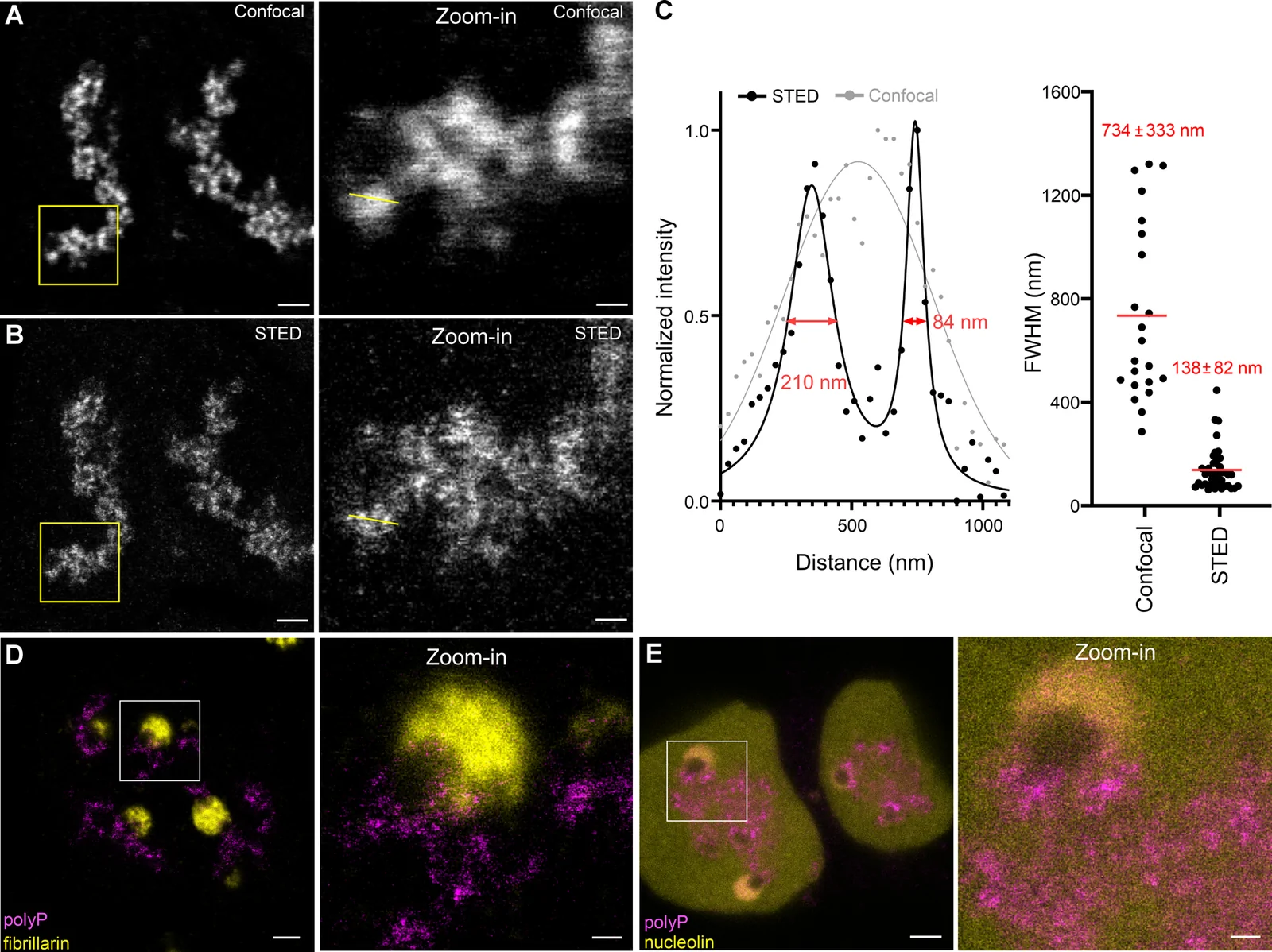
Subcellular distribution of polyP visualized using STED microscopy. (**A**) Representative confocal image of TREx-PPK1 cells induced with doxycycline for 24 h and stained with SiX-DPA-Zn (5.0 μM). Right panel: zoomed-in view of the region indicated by the yellow rectangle. (**B**) Corresponding STED images of the same nucleus. Right panel: zoomed-in view of the region indicated by the yellow rectangle. Scale bars: 2 μm and 500 nm (zoomed images). (C) Line profile plots of signal intensity along the yellow line in (**A**) and (**B**). Right graph: estimation of full width at half maximum (FWHM) of polyP granules from confocal (n = 22) and STED (n = 40) images from 20 nuclei. (**D, E**) Two-color STED imaging of polyP in TREx-PPK1 cells stained with SiX-DPA-Zn (5.0 μM, magenta), together with immunolabeled fibrillarin (**D**) or nucleolin (**E**) (yellow). Enlarged views on the right show the regions indicated by white squares. Scale bars: 2 μm and 500 nm (zoomed images).

The spatial distribution of polyP relative to nucleolar proteins was highly heterogeneous in the cell population (Figures 6D, E, S24 and S25), likely reflecting various degrees of nucleolar disorganization. Fibrillarin was detected in similar spherical compartments (Figures 6D and S27). These observations are consistent with previous reports showing that elevated polyP levels induce collapse of the nucleolus into round, homogeneous structures.^32^ Two distinct staining patterns were frequently observed in nucleolin. In cells with relatively weak SiX-DPA-Zn staining, polyP signals often colocalized with nucleolin. Both signals displayed a granular morphology characteristic of nucleolar substructures (Figure S28). In contrast, cells with strong polyP staining contained large and brightly labeled polyP aggregates with a similar granular appearance (Figures 6E and S29). In these cells, nucleolin was distributed diffusely throughout the nucleoplasm. In some cases, it accumulated in homogeneous spherical structures that did not overlap with polyP-positive regions and instead appeared spatially separated from them (Figures 6E and S29). Notably, no internal organization could be resolved within these compartments, even by STED microscopy (Figures 6D, E, and S27–29). The previous study also reported elevated phosphorus content in these rounded nucleolar structures, suggesting the presence of polyP.^32^ However, we did not observe strong SiX-DPA-Zn staining within such compartments. A possible explanation is that SiX-DPA-Zn does not efficiently detect polyP when it is highly complexed with metal ions, as observed in our *in vitro* experiments (Figure S11). This suggests that SiX-DPA-Zn visualizes a specific, accessible fraction of intracellular polyP. Together, these results establish SiX-DPA-Zn as a useful probe for investigating the relationship between polyP accumulation and nucleolar organization.

## Conclusion

In this study, we developed SiX-DPA-Zn, the first far-red fluorogenic chemosensor for selective detection of inorganic polyphosphates (polyPs) *in vitro* and *in cellulo*. The probe exhibits a strong preference for inorganic polyP over other abundant phosphate-containing molecules: ATP, ADP, GTP, CTP, UTP, DNA, and RNA. We demonstrated the applicability of SiX-DPA-Zn for polyP detection in solutions, yeast cell extracts, polyacrylamide gels, and fixed cells, where, for the first time, we were able to image polyP using super-resolution fluorescence microscopy. In Table S6, we provide a detailed comparison of SiX-DPA-Zn and other commonly used dyes in polyP research.

In solution experiments, the SiX-DPA-Zn signal is not affected by contaminating RNA, which is important when analyzing polyP from natural sources. In polyacrylamide gels, SiX-DPA-Zn detects polyP chains as short as n=3, enabling analysis of short polyPs and unambiguous assignment of their chain length. In addition, it offers a fast and simple staining procedure without extensive washing or prolonged and difficult-to-standardize UV irradiation. Most importantly, SiX-DPA-Zn can stain polyP in fixed cells, thereby opening the way for analysis of polyP levels and spatial distribution by fluorescent microscopy, including super-resolution STED imaging. Combined with immunostaining, it can become a valuable tool for the analysis of processes involving polyP in cells. Moreover, SiX-DPA-Zn is compatible with fluorescence-activated cell sorting (FACS), enabling the rapid and quantitative analysis of polyP levels in large cell populations.

A key objective for future probe development is the detection of polyP in live mammalian cells. Achieving this will require further optimization of the molecular design of SiX-DPA-Zn to improve its cell membrane permeability while preserving its polyP-detection capability.

Together, these features establish SiX-DPA-Zn as a broadly applicable tool for integrated quantitative and spatially resolved studies of polyP biology.

## Supplementary Material





## Notes

The Internet at Experimental methods, including chemical synthesis procedures; additional experimental details; supplementary Figures S1–S29; Tables S1–S6; copies of NMR spectra for compounds 1–8, SiX-DPA, and SiX-DPA-Zn (PDF)
Video S1: 3D polyP distribution inside the nucleus of an induced TREx-PPK1 cell (AVI)

## ‡

Bioinorganic Chemistry Laboratory, Department of Chemistry, Graduate School of Science, Nagoya University, Furo-cho, Chikusa-ku Nagoya, Aichi 464–8602, Japan

## Abbreviations

polyP: polyphosphate
STED: stimulated emission depletion.
